# *Tetrameranthus* (Annonaceae) revisited including a new species

**DOI:** 10.3897/phytokeys.12.2771

**Published:** 2012-04-19

**Authors:** Lubbert Y.T. Westra, Paul J. M. Maas

**Affiliations:** 1 Netherlands Centre for Biodiversity Naturalis (section NHN), Biosystematics Group, Herbarium Vadense, Wageningen University, Generaal Foulkesweg 37, 6703 BL Wageningen, The Netherlands

**Keywords:** Annonaceae, taxonomy, Neotropics, *Tetrameranthus*

## Abstract

The taxonomic revision of the infrequently collected genus *Tetrameranthus* by Westra (1985) is updated. A new species is described from French Guiana and Amapá, Brazil, increasing the number of species in this genus to seven.

## Introduction

An as-yet unknown species of the rare genus *Tetrameranthus* has been collected a few times in French Guiana and also once in neighboring Amapá, Brazil. The material was too incomplete to justify publication. Recently a new collection from French Guiana came in bearing ripe fruits and also reported to have a white flower. This made us decide now to formally publish this new species. We then also decided to update the revision of the genus *Tetrameranthus* (Westra, 1985), with the inclusion of *Tetrameranthus globuliferus* published later (Westra, 1988), adding the present new species as well. Some, mostly color, photographs of this seldom collected genus serve as an illustration.

## Taxonomic history

The Annonaceae form a large pantropical family, the largest family in the order Magnoliales, currently with (about) 129 genera and >2000 species (Stevens 2001 onwards). In the Neotropics the family is represented by 40 genera and c. 900 species (Chatrou et al. 2004). Annonaceae are woody plants, mostly trees and shrubs; lianas are mainly found in the Old World, only few lianas occur in the New World. The family is very distinctive and generally easy to recognize by, among others, simple leaves placed in two rows and without stipules, flowers with perianth members in 3-merous whorls, many tightly packed stamens, and a varying number of free carpels that remain free or become fused in fruit.

The Neotropical genus *Tetrameranthus* was described by Fries (1939) with the only species *Tetrameranthus duckei* R.E.Fr., It is aberrant by, among others, the unusual leaf disposition in a spiral instead of in two rows, the presence of four bracts placed in a whorl under the articulation of the flower stalk, and, above all, the perianth consisting of 4 sepals and 2 whorls of 4 petals each. The position of this peculiar genus is discussed by Fries in that paper, placing *Tetrameranthus* in an isolated position within the Annonaceae.

Over fifteen years later Fries added a second species, *Tetrameranthus macrocarpus* R.E.Fr., from Southeastern Colombia (Fries 1957). In his large survey of the whole family Fries even more emphasized the isolated position in placing it as the only member in a tribe Tetramerantheae in subfamily Annonoideae (that also included Uvarieae and Unoneae) (Fries, 1959).

Four more species were added in later years, viz. *Tetrameranthus laomae* D.R.Simpson from Eastern Peru (Simpson 1975), *Tetrameranthus pachycarpus* Westra from the environs of Iquitos, Peru, *Tetrameranthus umbellatus* Westra from northern Amazonian Peru, and *Tetrameranthus globuliferus* Westra from Yasuní National Park, Ecuador (Westra 1985, 1988). Gradually more material was collected, particularly around Manaus (*Tetrameranthus duckei*), and near Iquitos (*Tetrameranthus pachycarpus*). Outside these areas of concentration mainly collections from scattered localities have come in spanning a range from Andean Amazonia and the Pacific coast of Colombia (Chocó: one collection) in the West to French Guiana and adjacent Amapá, Brazil, in the East – including now the newly described *Tetrameranthus guianensis* in this paper. With the notable exception of the locally common *Tetrameranthus duckei*, *Tetrameranthus* as a whole still must be regarded as a rather rare genus.

Based on recent molecular data it was shown that *Tetrameranthus* belongs to an early branching lineage within Annonaceae (Richardson et al. 2004; Couvreur et al. 2011; Chatrou et al. in press, in press). It is classified in the subfamily Ambavioideae Chatrou, Pirie, Erkens and Couvreur (Chatrou et al. in press). Within this subfamily the South American genus *Tetrameranthus* is part of a highly supported clade together with the Southeast Asian genus *Mezzettia* and the African genera *Ambavia* and *Cleistopholis*. Recent analyses show *Tetrameranthus* in a fully resolved clade as sister to the other three above mentioned genera (Surveswaran et al. 2010; Chatrou et al. in press).

The generic name is composed of the Ancient Greek word elements “tetra” (four), “meros” (part), and “anthos” (flower), referring to the four-parted flowers.

## Morphology

### Vegetative part

*Tetrameranthus* isa genus of shrubs to (large) trees. As for all Annonaceae, the leaves are simple, entire, pinnately veined, symmetrical, and lack stipules, generally of moderate to fairly large size (greatest length ±30 cm). In contrast to the rest of Annonaceae, where the leaves are in two alternate rows almost without exception (Fries 1959: p. 8), in all species of *Tetrameranthus* the leaves are spirally arranged. Another uncharacteristic feature of this genus is the apical disposition of the leaves, generally found 10 cm from the apex of the branches, the ones below that are shed.

This rosette-like appearance of leaf disposition is suggestive of a number of other plant families such as Sapotaceae, but not of Annonaceae. This could explain in part why *Tetrameranthus* might seem rare, i.e., simply because many botanists do not identify it as Annonaceae.

### lnflorescences

Inflorescences in *Tetrameranthus* are truly axillary. As for the structure of the inflorescence in Annonaceae in general, the reader is referred to publications by Fries (1919, 1959). *Tetrameranthus* conforms to Fries’s type 1 (1959: p. 13) in that it has a primary flower stalk which carries bracts below the articulation, but no bracts above it. Seen more closely, the inflorescence structure is somewhat unusual within Annonaceae. Single-flowered inflorescences, as most commonly seen in *Tetrameranthus*, appear as an articulate stalk bearing the flower. The articulation in all but one species is at some distance above the base (the leaf axil; see e.g. Figures 3A, 3E). The only exception is in *Tetrameranthus laomae* where the articulation is found at the base (see Figure 2E). Immediately below the articulation there are (mostly) 4 bracts in a whorl. The bracts are shed before or (shortly) after flowering. In *Tetrameranthus duckei* a flower is sometimes seen originating from the axil of one the bracts, thus creating a 2-flowered inflorescence (Figure 2A). The pedicel of that lateral, or second-order, flower has an articulation at the base and lacks bracts. In *Tetrameranthus umbellatus* all four bracts have the potential to develop a similar axillary flower, thus resulting in an umbel-like inflorescence (Figure 2G). Pedicels of lateral flowers being bractless, inflorescences of *Tetrameranthus* are static: they cannot expand by reiterative growth processes as is characteristic for the rhipidium in most Annonaceae. The inflorescence of *Tetrameranthus* is best circumscribed as a botryoid (Weberling and Hoppe 1996: p. 32–33), albeit mostly reduced to a single flower.

For these reasons the basal part of the first-order flower stalk up to the articulation and including the whorl of bracts is referred to as peduncle, while the part above the articulation is termed pedicel. The stalks of second-order flowers, then, are termed pedicels in their entirety.

### Flowers

**Figure 1.**
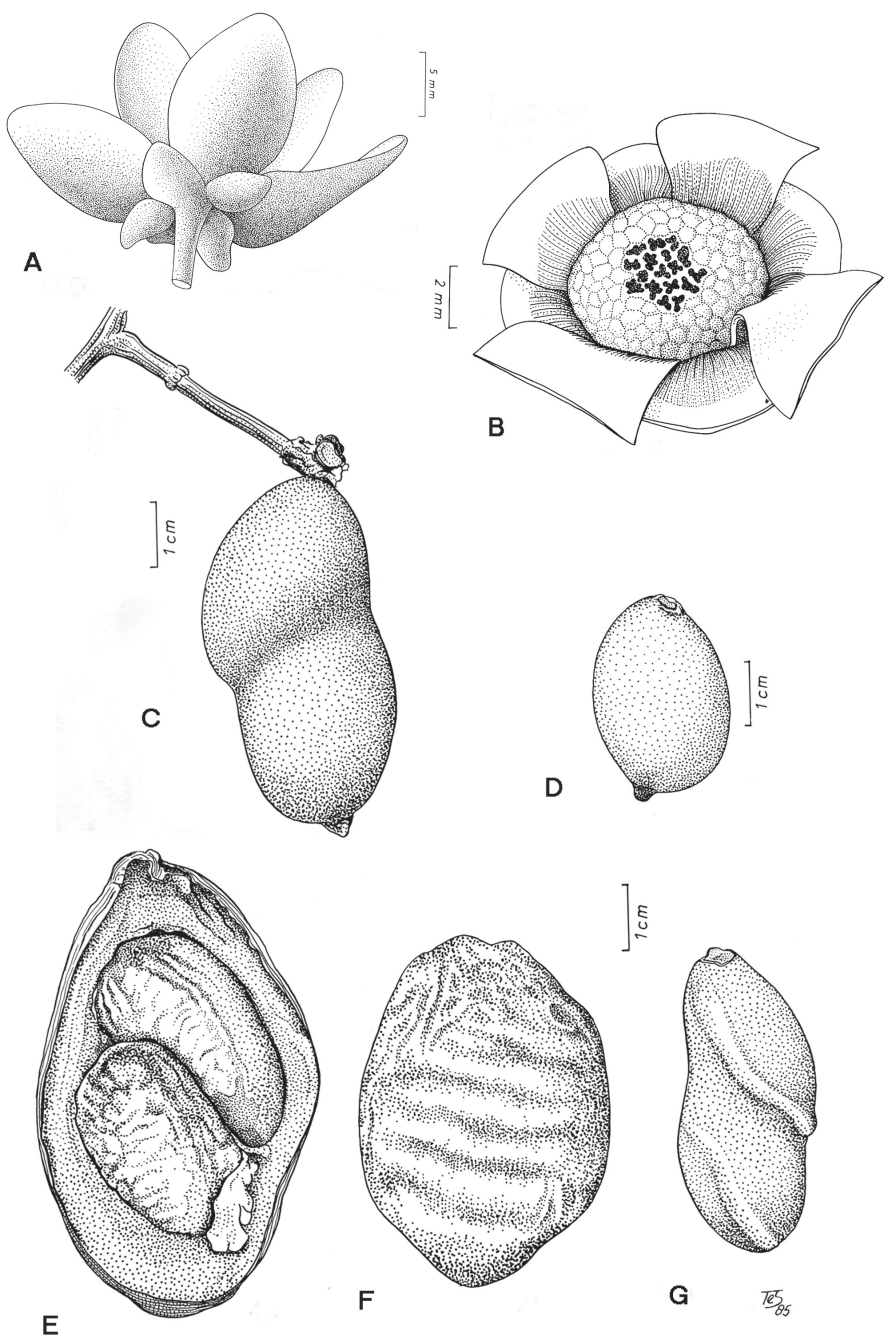
*Tetrameranthus umbellatus* Westra **A** Young flower (*Tunquí 62*, U) **B** Androecium and gynoecium of same. *Tetrameranthus duckei* R. E. Fr. **C** Two-seeded monocarp (*Rodrigues & Coêlho 3835*, U) **D** One-seeded monocarp (*Morawetz et al. 21-9883*, U). *Tetrameranthus pachycarpus* Westra **E, F** Two-seeded monocarp cut open and seen from outside (*Foster 4271*, NY). *Tetrameranthus umbellatus* Westra **G** Two-seeded monocarp (*Huashikat 613*, U).

As in most Annonaceae, the perianth consists of one whorl of sepals and two whorls of petals (Figure 1A). The whorls are 4-merous in at least five of the seven species, although incidentally a single deviating flower might occur, e.g. a 3-merous flower on one specimen of *Tetrameranthus laomae*, and a 5-merous flower on the type collection of *Tetrameranthus pachycarpus*. Such phenomena are not unusual throughout flowering plants in general, and have been observed in other Annonaceae genera (Couvreur 2009). *Tetrameranthus globuliferus* appears exceptional by having 6-merous whorls as far as seen, but this may need confirmation from more collections yet to be made. No good flower has been collected in *Tetrameranthus guianensis* so far, but see under the notes with that species. It should be stressed that in all other genera where deviations from trimery occur, this is an autapomorphy for individual species. In *Tetrameranthus*, however, this is synapomorphic for the genus (see also Saunders 2010).

The sepals are small in relation to the petals. They are free or connate just at the base. The aestivation is imbricate, only observable in very young buds as the sepals soon spread. The sepals drop after flowering.

**Figure 2.**
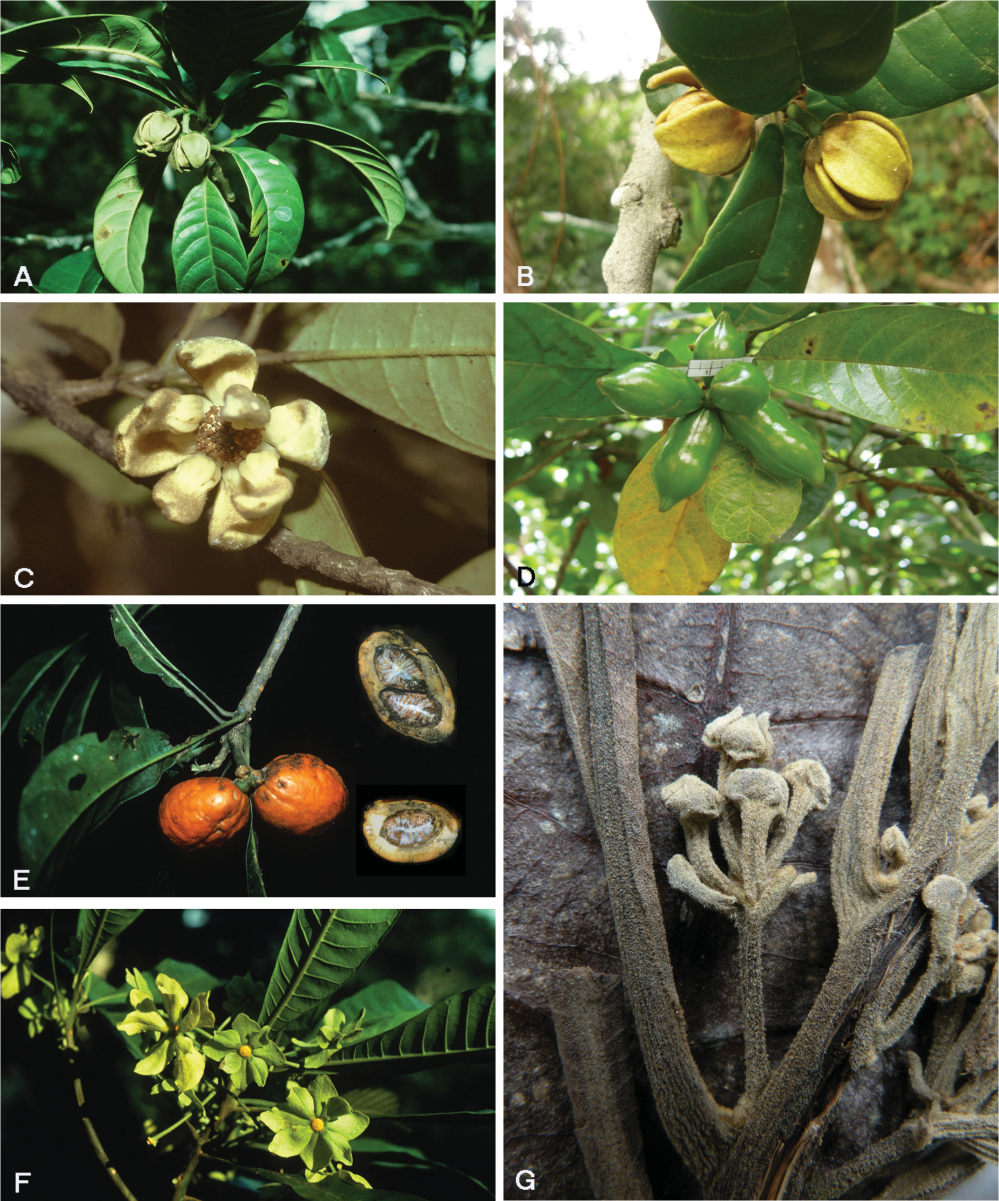
*Tetrameranthus duckei* R. E. Fr. **A** Two-flowered inflorescence **B** Flowers at early anthesis **C** Flower at late anthesis, liberation of pollen (Webber, 1981) **D** Fruit. *Tetrameranthus laomae* D.R.Simpson **E** Ripe fruit, also sectioned to show seeds. Note articulation at base of fruiting stalk. *Tetrameranthus umbellatus* Westra **F** Flowers **G** Young inflorescence, detail of herbarium specimen (*Morawetz & Wallnöfer 14-81085*, U). **A, E–F** Photos by W. Morawetz, **B–D** Photos by A. C. Webber.

The petals vary from rather fleshy (e.g. *Tetrameranthus duckei*, Figure 2C) to rather thin (*Tetrameranthus laomae*, *Tetrameranthus umbellatus*, Figure 2F),those of the outer whorl being somewhat larger or broader than those of the inner whorl. The photographs also showcurved petals in *Tetrameranthus duckei* in the living condition, while in *Tetrameranthus umbellatus* (and possibly also in *Tetrameranthus laomae*) the petals appear rather flat at least before anthesis. The petals are adorned with a callus or callus-like tissue at the base on the inner side: this is an area of varying size, depending upon the species, which is devoid of indument. In *Tetrameranthus duckei* the callus appears as a protruding hump (Figure 2C), particularly on the inner petals where it is even larger than on the outer petals. In the other species the callus is smaller in relation to the size of the whole petal than in *Tetrameranthus duckei*.

The convex torus bears mostly numerous stamens and a mostly rather small number of free carpels in the center. The stamens have a short filamental part, a thick connective capped by a massive, more or less conical or flat shield, and an extrorse to latrorse anther. The carpels contain two (exceptionally three), lateral, superposed ovules, and have on top a sessile stigma which varies from trilobed to an irregularly lobed disc.

Curculionidae beetles have been observed as pollinators in *Tetrameranthus duckei* (Webber 1981; Gottsberger 1999). There are no reports known to us so far for other *Tetrameranthus* species.

### Fruit

Depending on the species, the number of free monocarps varies from 1–15. The ellipsoid to oblongoid monocarps are fleshly, indehiscent, and two-seeded or, due to abortion of one ovule, one-seeded (rarely three seeds develop) (Figure 1C–G). The seeds are laterally attached, with the lowest one near the base, and are ascending, thus resulting in the characteristic oblique constriction seen in monocarps with >1 seed on herbarium specimens. In fresh fruits the constriction is less obvious, and in very thick-walled monocarps of some of the species (e.g. *Tetrameranthus guianensis*, *Tetrameranthus pachycarpus*) it becomes practically indistinguishable. In herbarium material the fruit wall is smooth in most species, but becomes shriveled in *Tetrameranthus globuliferus* (Westra 1988: pp 269, 278, Fig. 21) and *Tetrameranthus guianensis*.

The seeds are quite large in relation to the fruit body; they are slightly compressed dorsiventrally, and possess ruminations in the shape of fairly numerous lamellae protruding from the seed coat into the interior almost to the middle.

### Indument

The indument of *Tetrameranthus* consists of stellate hairs with 2–10 rays, varying with the species. In addition to stellate hairs, simple hairs are present in varying densities, also depending upon the species. Most genera of Neotropical Annonaceae have simple hairs, or have stellate hairs beside simple hairs in a small percentage of the species only (e.g. *Annona* including *Rollinia*). The notable exception (apart from *Tetrameranthus*) is *Duguetia*: in this genus most species even have scales, rather than stellate hairs (Maas et al. 2003).

Indument in *Tetrameranthus* isfound especially on young vegetative parts and inflorescences. Some of it persists on the primary vein of the leaves, and to a lesser extent on the secondary veins, mainly on the lower side; it is also seen on petioles and branchlets in the leafy zone. In *Tetrameranthus umbellatus* stellate hairs are also found diffusely spread over the leaf surface, especially on the abaxial side. In *Tetrameranthus laomae*, too, scattered stellate hairs may be spotted on the lower leaf surface.

Floral parts, with the exception of stamens and the callus area on petals mentioned earlier, are usually covered with a dense indument of stellate hairs. Carpels, when enlarging into monocarps, quickly become glabrous.

The trichome length on vegetative parts (except in a very young stage, and persisting near axils) does not exceed 0.1–0.2 mm in *Tetrameranthus laomae* and *Tetrameranthus umbellatus*: these two species thereby are easily distinguished from the other ones, where considerably longer (to 0.5 mm, or even more) and stiffer trichomes are found, next to simple hairs of the same size. Trichomes on inflorescences and flower parts may reach a somewhat larger average size than those on vegetative parts (this we did not investigate in detail).

## Taxonomic treatment

### Genus Tetrameranthus

R.E.Fr.

http://species-id.net/wiki/Tetrameranthus

Tetrameranthus Acta Horti Bergiani 12(3): 554. fig. 41. 1939; Westra, Proceedings of the Koninklijke Nederlandse Akademie van Wetenschappen, ser. C.88: 449–482. 1985.

#### Type.

*Tetrameranthus duckei* R.E.Fr.

#### Description.

*Trees* or *shrubs*. Leafy twigs and most floral parts sparsely to densely covered with stellate to simple hairs to glabrous. *Leaves* spirally arranged, often concentrated towards the end of the branches, primary vein impressed to slightly raised on the upper side. *Inflorescences* axillary, 1-flowered to several-flowered and umbel-like, bracts up to 4 below the articulation. *Flowers* bisexual, 4-merous or less often 5–6-merous, white to yellow or cream; sepals 4(–6), imbricate, free or basally connate; petals 8(–12), free, subequal, imbricate, much longer than the sepals, often with a callus at the inner base; stamens numerous, connective shield discoid, either flat, cushion-shaped, or with a conical prolongation; carpels c. 5–30, ovules 1–2(–3), lateral, stigma sessile, more or less lobed. *Fruit* apocarpous; monocarps 1–15, free, sessile or sometimes narrowed into a short and thick stipe-like base, indehiscent, sometimes constricted, wall rather thick (1–7 mm) and fleshy. *Seeds* 1–2(–3) per monocarp, lower one near the base, upper one(s) lateral.

#### Distribution.

Seven species in the Amazon regions of Venezuela, Colombia, Brazil, Peru, and Ecuador, but also in the Colombian state of Chocó and in French Guiana and neighboring Amapá, Brazil.

#### Key to the species of Tetrameranthus

**Table:** 

la	Young plant parts covered with stellate hairs ≤0.2 mm long; primary vein mostly flat (or slightly raised or slightly impressed) above	2
1b	Young plant parts covered with stellate and simple hairs ≥0.5 mm long; primary vein impressed (exceptionally almost flat) above	3
2a	Inflorescences with up to 5 umbellately arranged flowers; peduncle manifest, ≥5 mm long (Amazonian Peru and Brazil)	*Tetrameranthus umbellatus*
2b	Inflorescences 1-flowered; peduncle inconspicuous, <1 mm long. (Amazonian Peru and Brazil and the Colombian states of Amazonas and Chocó)	*Tetrameranthus laomae*
3a	Monocarps globose or almost; perianth (as far as known) of 6-merous whorls (Amazonian Ecuador)	*Tetrameranthus globuliferus*
3b	Monocarps ellipsoid, oblongoid or fusiform; perianth of 4-merous or less often 5-merous whorls	4
4a	Monocarps, both 1- and more-seeded ones, ellipsoid or fusiform, without constriction or with a weak constriction	5
4b	2-Seeded monocarps oblongoid, with a manifest oblique constriction about the middle	6
5a	Monocarps 7–15, 35–60 by 20–30 mm, wall shriveled in sicco; petioles ≤10 mm long (French Guiana and the Brazilian state of Amapá)	*Tetrameranthus guianensis*
5b	Monocarps 1–3, to c. 70 by 40 mm, wall not shriveled in sicco; petioles ≥20 mm long (Amazonian Peru, vicinity of Iquitos)	*Tetrameranthus pachycarpus*
6a	Monocarps ≥35 mm in diam.; tall tree (Amazonian Colombia)	*Tetrameranthus macrocarpus*
6b	Monocarps ≤25 mm in diam.; shrub or small tree ≤12 m (Amazonian Colombia, Venezuela, and Brazil)	*Tetrameranthus duckei*

### Tetrameranthus duckei

R.E.Fr.

http://species-id.net/wiki/Tetrameranthus_duckei

**Map 1.**
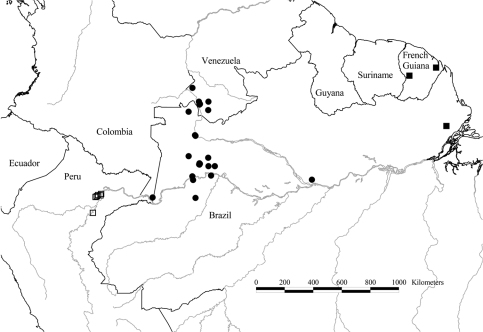
Distribution of *Tetrameranthus duckei* R. E. Fr. (●), *Tetrameranthus guianensis* Westra & Maas (■), and *Tetrameranthus pachycarpus* Westra (□).

Figs 1C, D 2A–D Map 1

Tetrameranthus duckei Acta Horti Bergiani 12(3): 557. 1939.

#### Type.

*Ducke RB 23919* (holotype S; isotypes RB, S), Brazil, Amazonas: Manaus, Estrada do Aleixo, km 7, 14 June 1933.

#### Description.

*Shrub* or *tree*, 3–12 m tall, 4–8 cm diam., young twigs and petioles densely to rather densely covered with brown, stellate hairs >0.5 mm long, becoming glabrous. *Leaves*: petioles 10–40 mm long, 1.5–4 mm diam.; lamina narrowly elliptic to narrowly obovate, 10–25 by 3–10 cm (index 2.7–4), chartaceous to coriaceous, dull or slightly shiny brown or greenish brown above, dull brown or greenish brown below in sicco, rather densely covered with stellate hairs on primary vein, otherwise glabrous above, rather densely to sparsely covered with stellate hairs on primary vein and secondary veins, otherwise mostly glabrous below, the stellate hairs similar to those on branchlets, base acute, apex acuminate (acumen 5–25 mm long), primary vein impressed above, secondary veins 8–12 on either side of primary vein, impressed above, loop-forming, shortest distance between loops and margin 1.5–5 mm, or not loop-forming in basal part, tertiary veins slightly raised, flat, or indistinct above, percurrent to reticulate. *Inflorescences* 1(–2)-flowered, peduncles 5–15 mm long, c. 1.5 mm diam., fruiting peduncles to c. 3 mm diam., bracts 4, narrowly triangular, 3–6 mm long, soon falling after flowering, pedicels 10–25 mm long, c. 1.5 mm diam., fruiting pedicels to c. 40 mm long, 3 mm diam., peduncles and pedicels densely covered with stellate hairs, becoming glabrous. Flowers green, turning yellow in vivo; sepals elliptic to obovate, free, 5–7 mm long, outer side densely covered with stellate hairs; outer petals ovate, 20–25 by 9–12 mm, inner base with fleshy and longitudinally grooved callus 5–6 mm long and extending across the whole width, inner petals narrowly ovate to ovate, 15–22 by 6–9 mm, with similar callus to c. 8 mm long, outer side of petals densely covered with stellate hairs, the callus on the inner side glabrous; stamens 2–2.5 mm long, connective shield conical or acuminate, 1–1.5 mm long, more or less curved toward the center. *Monocarps* 1–6, green or shiny green, turning green-yellow in vivo, brown to dark brown in sicco, ellipsoid or oblongoid to narrowly so, 25–65 by c. 20(–25) mm, with (2-seeded forms) or without oblique constriction, apex a thick obtuse beak 2.5–10 mm long. *Seeds* 1–2 per monocarp, to c. 35 by c. 20 mm.

#### Distribution.

Amazonian regions of Venezuela (Amazonas), Colombia (Guainía), and Brazil (Amazonas, most common in Manaus and vicinity).

#### Habitat and ecology.

Mostly in low forest or shrub vegetation (campina, campinarana, Amazonian caatinga, and bana) on white sand. At low elevations up to c. 200 m. Flowering and fruiting: throughout the year.

#### Additional specimens examined.

**Colombia**. Guainía: Puerto Colombia (opposite Venezuelan town of Maroa) and vicinity, alt. 800–850 ft, Schultes et al. 18157 (US).**Venezuela**. Amazonas: Mun. Guainía, along road from Maroa to Yavita, Acevedo-R. et al. 10250 (U); Lower Río Guainía, Raudal Lombriz, 2 km from mouth of Río Casiquiare, 140 m, Aymard et al. 9751 (MO, U); Río Casiquiare, below Capibara (“Capihuara”), Colella et al. 1879 (U); Río Casiquiare, El Porvenir, Colella et al. 2170 (U); San Carlos de Río Negro, Christenson 1386 (US), Liesner 6744 (MO, U), 7590 (MO, U), 8598 (MO, U), 8838 (MO, U), Steyermark & Bunting 102721 (NY, US); Río Negro, base of Piedra de Cucuy, 100–200 m, Maas et al. 6879 (INPA, NY, U); Río Pasimoni, 80 m, Velazco 1954 (MO, U). **Brazil.** Amazonas: Manaus and vicinity, Almeida INPA 3580 (INPA), L. Coêlho INPA 3673 (INPA, S), Ducke 1908 (F, NY), Ducke RB 35313 (RB, S), Ferreira 79/57 (S), Miralha et al. 230 (INPA, U), Morawetz et al. 21-9883 (WU), 21-23883 (WU), 22-19883 (WU), 24-12983 (WU), Personel of Centro de Pesquisas Florestais INPA 6232 (INPA, S), Plowman et al. 12647 (U), Prance et al. 2721 (INPA, NY, US), 3816 (NY, US), 4679 (NY, US), Rodrigues & L. Coêlho 2937 (U), Rodrigues & Almeida 3068-A (U), Rodrigues & Lima 3454 (U), Rodrigues & D. Coêlho 3839 (INPA, U), Rodrigues 8742 (INPA), Webber 162 (U), 163 (U); Reserva Florestal Ducke, Igarapé Acará, Ribeiro et al. 1501 (INPA),1749 (INPA, U), Sothers et al. 757 (INPA, U); Rio Negro, at its confluence with Rio Vaupés, Serra Canaleão, 150 m, Stevenson et al. 1002 (NY, U); Rio Javari, behind Estirão de Equador, Lleras et al. P17302 (NY, U); mouth of Rio Vaupés, Pires et al. 7473 (S).

#### Vernacular names.

Venezuela: Banayo (*Liesner 7590*), Cuchara (*Liesner 6744*), Majagua (*Velazco 1954*), Palo de cuchara (*Liesner 7590*). Brazil: Envira (*Ferreira 79/57*).

#### Note.

In a previous paper (Westra, 1985) there was some doubt about the identity of the collection *Lleras et al. P17302*. It should be regarded as no more than an extreme form of *Tetrameranthus duckei*, with pedicels to c. 40 mm long and outer petals to c. 15 mm wide.

### Tetrameranthus globuliferus

Westra in Maas et al.

http://species-id.net/wiki/Tetrameranthus_globuliferus

Fig. 3D–G Map 2

Tetrameranthus guianensis Proceedings of the Koninklijke Nederlandse Akademie van Wetenschappen, ser. C. 91: 262, figs 20–22. 1988.

#### Type.

*Lawesson et al. SEF 8779* (holotype AAU; isotypes AAU, QCA, QCNE, U), Ecuador, Orellana: Añangu, Parque Nacional Yasuní, 260–350 m, May–June 1986.

#### Description.

*Medium-sized tree*, >10 cm diam., young twigs and petioles densely covered with pale brown, stellate hairs >0.5 mm long. *Leaves*: petioles 4–8 mm long, 4–6 mm diam., lamina narrowly obovate, 27–37 by 9–15 cm (index 2.8–2.9), chartaceous, shiny green above in vivo, greenish brown above, pale greenish brown to brown below in sicco, rather densely covered with stellate hairs >0.5 mm long on primary vein, to rather sparsely so on smaller veins on both sides, base acute to attenuate, to obtuse or rounded at the extreme base, apex acute to acuminate (acumen to c. 10 mm long), primary vein flat to slightly raised above, secondary veins 20–25 on either side of primary vein, flat to impressed above, loop-forming, shortest distance between loops and margin 1.5–3 mm, or not loop-forming, tertiary veins flat to raised above, percurrent to more or less reticulate. *Inflorescences* 1-flowered; peduncles 3–5 mm long, 3–4 mm diam., fruiting peduncle c. 5 mm diam., bracts [4?] narrowly oblong or narrowly triangular, 4–5 mm long, falling after flowering, pedicels {18–30} mm long, {4–6} mm diam., densely covered with brownish, stellate hairs; flowers with perianth in 6-merous whorls, cream with the inner petals yellow at the inner base in vivo; sepals broadly ovate-triangular, connate at the very base, {6–11} by {5–10} mm; outer petals elliptic to ovate, {30–45} mm long, {10–25} mm wide, with small callus at the inner base; inner petals {30–40} mm long, {5–10} mm wide, more or less narrowed toward the base, with larger callus, outer side of petals densely covered with stellate hairs, the callus on the inner side glabrous; stamens {2–2.5} mm long, connective shield flat, cushion-shaped. *Monocarps* 2–7, globose or almost, green in vivo, brown in sicco, c. 40 mm diam., wall strongly shriveled in sicco. *Seeds* 1–2 per monocarp, 25–30 by 15–20 mm.

**Figure 3.**
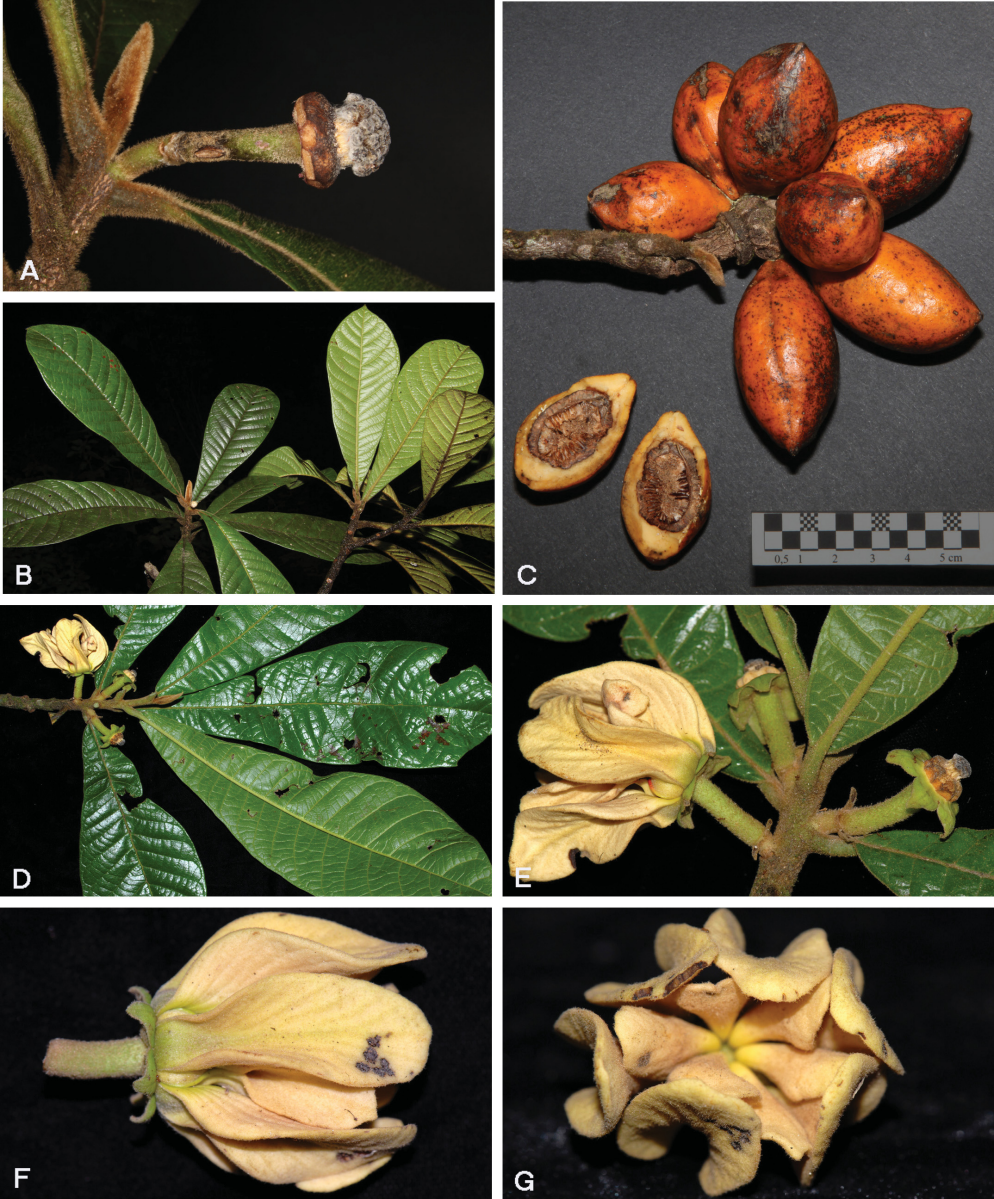
*Tetrameranthus guianensis* Westra & Maas **A** Single-flowered inflorescence after shedding of perianth and stamens **B** A twig **C** Ripe fruit, also sectioned to show seed. *Tetrameranthus globuliferus* Westra **D, E** Twig with inflorescences (*Pérez C. & Santillán 4404*, QCA) **F, G** Flower seen from the side and from above (same). **A–C** Photos by D. Sabatier, **D–G** Photos by A. J. Pérez C.

#### Distribution.

Ecuador (Orellana). Only known so far from Parque Nacional Yasuní.

**Map 2.**
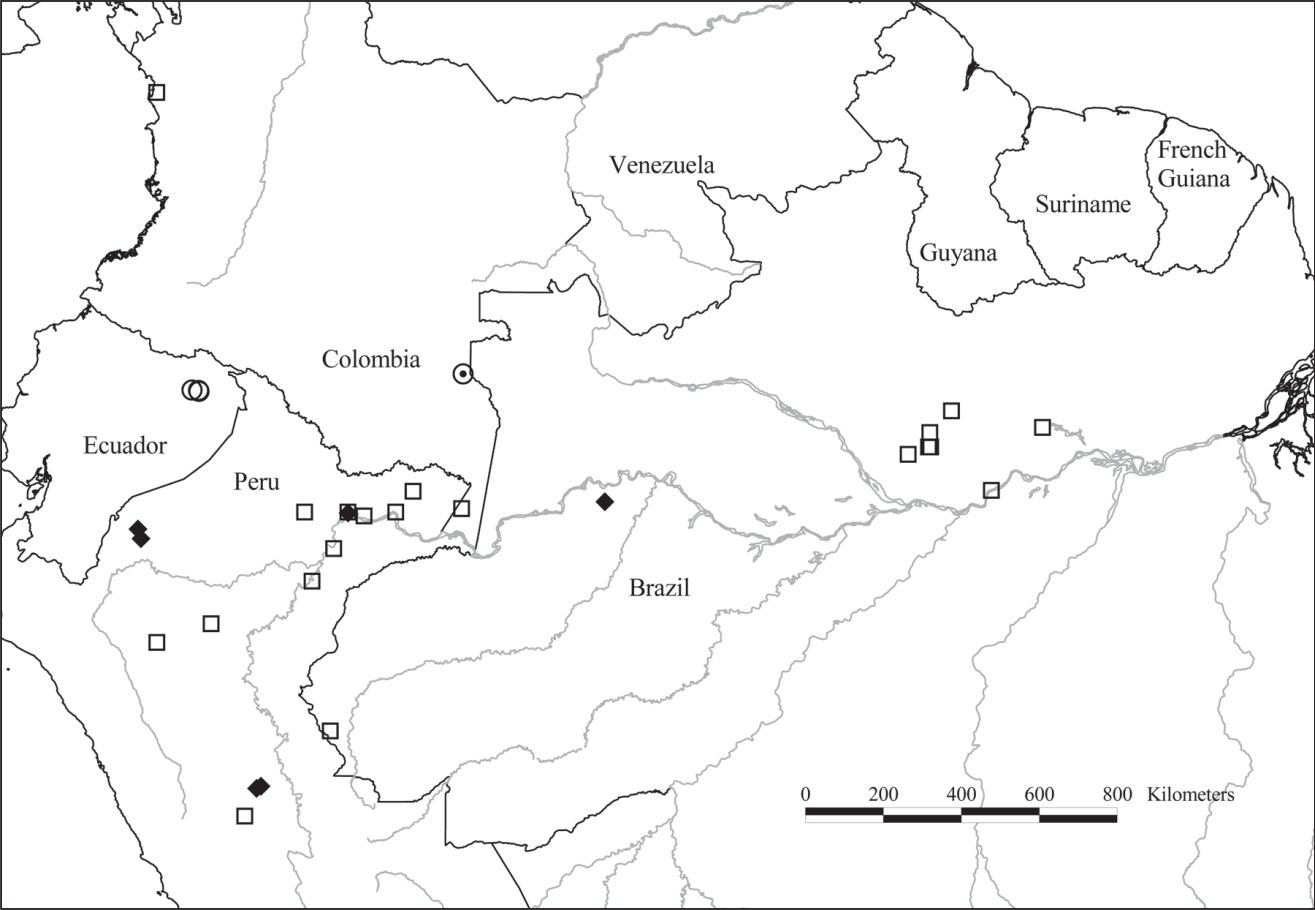
Distribution of *Tetrameranthus globuliferus* Westra (○), *Tetrameranthus laomae* D. R. Simpson (□), *Tetrameranthus macrocarpus* R. E. Fr. (◉), and *Tetrameranthus umbellatus* Westra (◆).

#### Habitat and ecology.

In rain forest on terra firme. At elevations of 200–400 m. Flowering recorded in November; fruiting recorded in May, June, August.

#### Additional specimens examined.

**Ecuador**. Orellana: NW corner of Parque Nacional Yasuní, 355–365 m, Korning & Thomsen 47626 (AAU, U); Parque Nacional Yasuní, E of Juan Tapuy’s finca, 250 m, Pitman & Delinks 1440 (MO, QCA,U).

#### Note.

The description of the flower was largely made from a very recent photograph of a freshly collected twig of *Pérez & Santillán 4404* (QCA). Measurements between { } were made on two flowers preserved in alcohol kept in QCA and were kindly supplied to us by Álvaro J. Pérez C.

Á. J. Pérez also reports to us a clustered occurrence of *Tetrameranthus globuliferus*: he found few individuals all close to the one that he collected, but did not spot any more around the trails of the Yasuní Scientific Station.

The species has been listed as near-threatened on the IUCN red list (Muriel and Pitman 2003). In an important paper on global conservation significance of Yasuní National Park *Tetrameranthus globuliferus* is documented as one of five species (and one of only two woody species) not found anywhere else in the world (Bass et al. 2010).

### Tetrameranthus guianensis

Westra & Maas sp. nov.

urn:lsid:ipni.org:names:77118748-1

http://species-id.net/wiki/Tetrameranthus_guianensis

Fig. 3A–C Map 1

#### Latin.

A speciebus ceteris hujus generis pilis stellatis vel simplicibus validis et monocarpiis rugosis in statu sicco distinctus.

#### Type.

*Sabatier & Gonzalez 5387* (holotype CAY; isotype L), French Guiana, Savane-roche Virginie, Parcelle SRV2, 4°11'N, 52°9'W, 18 March 2008.

#### Description. 

*Tree*, 8–25 m tall, 10–25 cm diam., slash yellow-orange (*Mori et al. 23521*); young twigs and petioles densely covered with brownish, stellate and simple hairs >0.5 mm long. *Leaves*:petioles 5–10 mm long, 3–4 mm diam., more or less thickened toward the base; lamina narrowly obovate to obovate-elliptic, 14–26 by 5–10 cm (index 2.4–2.9), chartaceous, shiny green above in vivo, dark brown above and pale brown below in sicco, rather densely covered mainly on large veins to rather sparsely covered with stellate hairs >0.5 mm long or glabrous elsewhere above, densely covered with stellate hairs on large veins, rather densely to sparsely so or almost glabrous elsewhere below, base acute to attenuate, apex abruptly acuminate (acumen 2–12 mm long), primary vein impressed above, secondary veins 14–19 on either side of primary vein, impressed above, loop-forming, shortest distance between loops and margin 1–3 mm, or not loop-forming, tertiary veins impressed above, percurrent to reticulate. *Inflorescences* 1-flowered, only seen in postfloral and fruiting stages, peduncles c. 10 mm long, c. 3 mm diam., fruiting peduncles to c. 5 mm diam., pedicels c. 20 mm long, c. 3 mm diam., fruiting pedicels to c. 5 mm diam., peduncles and pedicels densely covered with brownish, stellate and simple hairs; sepals not seen; petals whitish (fide collectore) in vivo, estimated to be c. 35 by 40 mm; stamens not seen. *Monocarps* 7–15, ellipsoid to fusiform, yellowish green to yellowish orange in vivo, pale brown in sicco, 35–60 by 20–30 mm, apex obtuse, rounded, or bluntly pointed, with or without weak oblique constriction, wall shriveled in sicco. *Seeds* 1–3 per monocarp, 25–30 by 15–20 by 15 mm.

#### Distribution.

French Guiana and the adjacent Brazilian state of Amapá.

#### Habitat and ecology.

In forests. At an elevation of c. 100 m. Flowering recorded in December; fruiting recorded in March, July, and December.

#### Notes.

*Tetrameranthus guianensis* is the first species of the genus reported from the Guianas. It is distinct from other species of *Tetrameranthus* by a dense cover of coarse stellate and simple hairs on all vegetative parts. Like the Ecuadorian *Tetrameranthus globuliferus*, it has shriveled fruit walls in dry condition.

As no complete flowers were available in herbarium material, description of floral characters is based in part on field observations of a single living flower at distance. The collector, D. Sabatier, has informed us that an attempt to collect that flower which was high up in a large tree and out of reach had failed. Sabatier (pers. comm.) notices 5 scars per whorl on the receptacle (compare Figure 3A) implying that we have a 5-merous flower here! This definitely requires confirmation from further collections, though.

Two sterile collections, namely Mori et al. 23521, 23674, also from French Guiana, seem to come quite near this species, differing mainly in the leaf shape (obovate-elliptic, rather than narrowly obovate), and the less dense and more coarse indument of stellate hairs of comparable size. It concerns trees of 8 m, 10 cm diam., and 15 m, 12 cm diam., respectively, from non-flooded moist forest. More material, and more complete in particular, is needed here.

#### Additional specimens examined.

**French Guiana**. Sinnamary River, above Petit Saut, between Crique Plomb and Crique Tigre, 500 m above Saut Tigre in area to be inundated by waters of Petit Saut Dam, Mori et al. 23521 (CAY, NY, U), Mori et al. 23674 (CAY, NY, U); Rivière Grand Inini, Basin of Maroni River, Arbre II-59, 3°40'N, 53°50'W, Sabatier & Prévost 3084 (CAY, P, U); Savane-roche Virginie, Parcelle SRV2, 4°11'N, 52°9'W, Sabatier 5784 (CAY, WAG). **Brazil.** Amapá: Rio Araguari, upland plant, Pires et al. 51490 (MG).

### Tetrameranthus laomae

D.R.Simpson

http://species-id.net/wiki/Tetrameranthus_laomae

Fig. 2D, E Map 2

Tetrameranthus laomae Phytologia 30(5): 309. 1975.

#### Type.

*Soria S. 64* (holotype F), Peru, Loreto: Alto Amazonas, Distr. Yurimaguas, road from Yurimaguas to Tarapoto, km 19 from Yurimaguas, 115 m, 11 March 1969.

#### Description.

*Tree*, 7–35 m tall, 11–45 cm diam., young twigs and petioles densely covered with whitish, stellate hairs <0.2 mm long, becoming glabrous. *Leaves*: petioles 10–20 mm long, 1–1.5 mm diam.; lamina obovate to narrowly obovate or narrowly elliptic-obovate, 6–24 by 2–7 cm (index 2.4–3.7), chartaceous to thinly coriaceous, shiny to dull greenish brown above, dull greenish brown below in sicco, rather densely to sparsely covered with stellate hairs <0.2 mm long on primary vein, otherwise sparsely so to glabrous on both sides, base attenuate, decurrent along petiole, apex acute or acuminate (acumen (0–)7–20 mm long), primary vein raised to almost flat above, secondary veins 7–10 on either side of primary vein, more or less raised above, loop-forming, shortest distance between loops and margin 2–4 mm, or not loop-forming in basal part, tertiary veins raised above, reticulate and often tending to form intersecondaries. *Inflorescences* 1-flowered, peduncles 0–1 mm long, bracts 3, narrowly triangular or narrowly oblong, 1–2.5 mm long, soon falling after flowering, pedicels 7–15 mm long, 1–1.5 mm diam., fruiting pedicels to c. 20 mm long, 2.5–4 mm diam., densely to rather densely covered with stellate hairs, becoming glabrous. *Flowers* green to yellowish, green with yellow center, or yellow in vivo; sepals broadly elliptic to broadly ovate, 2.5–3 by 2–2.5 mm, outer side densely covered with stellate hairs; outer petals ovate-elliptic, 10–16 by 5–8 mm, inner petals narrowly elliptic, 8–11 by 3–4 mm, outer side of petals rather densely covered with stellate hairs, the inner base glabrous; stamens c. 1 mm long, connective shield cushion-shaped, flat. *Monocarps* 4–13, green, maturing orange or red in vivo, black in sicco, ellipsoid to oblongoid, 2-seeded forms 30–45 by 15–<20 mm and with oblique constriction, 1-seeded forms smaller and without constriction, apex mostly rounded. *Seeds* 1–2 per monocarp, to c. 25 by 20 mm.

#### Distribution.

Amazonian Peru (Loreto, Pasco) and Brazil (Acre, Amazonas, Pará), and the Colombian states of Amazonas and Chocó.

#### Habitat and ecology.

In forest on terra firme, on clay or sandy soil. At low elevations to c. 350 m. Flowering and fruiting: throughout most of the year.

#### Additional specimens examined.

**Colombia.** Amazonas: Mun. Leticia, Corregimiento de Tarapacá, Parque Nacional Natural Amacayacu, Cabaña Pamaté, 100 m, Rudas et al. 2611 (MO, U). Chocó: Parque Nacional de Utría, 0–100 m, García C. & Agualimpia 398 (MO, U). **Brazil.** Acre: vicinity of Serra da Moa, Prance et al. 12236 (MO, NY, U). Amazonas: Mun. Itapiranga, Rio Uatumã, above confluence of Rio Uatumã with Rio Pitinga, Cid et al. 824 (MO, NY, U); Mun. Presidente Figueiredo, near Represa de Balbina, Cid et al. 6657 (NY, U), 8084 (NY, U), Thomas et al. 5271 (NY, U), 5358 (NY, U). Pará: Mun. Oriximiná, Rio Trombetas, Lago Maincoé, 8 km NE of Mineração Santo Patricia, 80 m, Martinelli 7338 (NY, U). **Peru.** Loreto: Guarnición Pijuayal, near Pebas, 130 m, Díaz S. et al. 571 (MO); Distr. Las Amazonas, “Roca Eterna”, 120–130 m, Grández & Jaramillo 2850 (MO, U); Distr. Las Amazonas, Explornapo Camp, near Sucusari, 100–140 m, Pipoly et al. 13386 (MO, U), 13407 (MO, U), 13496 (MO,U), 14575 (MO, U); Prov. Mariscal Ramón Castilla, Upper Río Yaguas, tributary of Río Putumayo, 80 km NE of Pebas, 140 m, Ríos et al. 464 (U); Prov. Ucayali, Sapuena, Jenaro Herrera, 130 m, Vásquez et al. 12012 (MO, U); Prov. Maynas, Sargento Lores, Esperanza, 120 m, Vásquez & Jaramillo 13249 (MO, U). Pasco: Prov. Oxapampa, Cabeza de Mono, Río Iscozacín, Palacazu Valley, 320 m, Gentry et al. 41743 (MO, U); Prov. Oxapampa, Distr. Iscozacín, Pariona & Ruíz C. 1038 (MO).

#### Vernacular names.

Peru: Sacha anona (*Pariona & Ruíz C. 1038*).

#### Uses.

Peru: used for construction work on the countryside (*Soria S. 64*).

### Tetrameranthus macrocarpus

R.E.Fr.

http://species-id.net/wiki/Tetrameranthus_macrocarpus

Map 2

Tetrameranthus macrocarpus Arkiv för Botanik Ser. 2, 3(18): 603, plate 4. 1957.

#### Type.

*Schultes & Cabrera 17091* (holotype S; isotypes COL, GH, U), Colombia, Vaupés: Río Piraparaná, Raudal Coro (“Koro”), 30 August 1952.

#### Description.

Tall *tree*, young twigs and petioles densely to rather densely covered with stellate hairs >0.5 mm long. *Leaves*: petioles 20–30 mm long, 3–4 mm diam., slightly thickened toward the base; lamina elliptic to obovate to narrowly so, 14–20 by 5–8 cm (index 2–2.9), coriaceous, glabrous above, rather densely to sparsely covered with stellate hairs on large veins, otherwise glabrous below, base acute to attenuate, apex acuminate (acumen 5–20 m long), primary vein impressed above, secondary veins 9–11 on either side of primary vein, impressed above, loop-forming, shortest distance between loops and margin 1–2 mm, or not loop-forming, tertiary veins flat and inconspicuous above, percurrent to reticulate. *Inflorescences* 1-flowered; peduncles c. 5 mm long, 2–2.5 mm diam., fruiting peduncles to c. 5 mm diam.; bracts 4, triangular to narrowly triangular, 2–3 mm long, outer side densely covered with stellate hairs, falling after flowering; pedicels 25–30 mm long, c. 2 mm diam., fruiting pedicels to c. 35 mm long, peduncles and pedicels densely covered with stellate hairs, becoming glabrous. *Flowers*: color not mentioned; sepals broadly triangular-ovate, connate just at the base, 3–4 mm long, outer side densely covered with stellate hairs; outer petals elliptic, 25–30 mm long, 12–15 mm wide, base attenuate into a more or less distinct claw, apex acute to obtuse, callus area at inner base to c. 5 mm long, inner petals similar to outer petals, 20–25 mm long, outer side of petals densely covered with stellate hairs; stamens c. 3 mm long, connective shield with conical prolongation (fide Fries 1957). *Monocarps* 1–2, green, probably maturing yellow in vivo, brown in sicco, ellipsoid or oblongoid, with (2-seeded forms) or without oblique constriction, apex rounded, to c. 60 (1-seeded) or c. 80 by 35 mm (2-seeded). *Seeds* 1–2 per monocarp, to c. 35 by 28 mm.

#### Distribution.

Colombia (Vaupés). Only known from the type collection.

#### Habitat and ecology.

Not recorded.

#### Note.

This single collection of *Tetrameranthus macrocarpus*seems to come quite near some forms of the variable *Tetrameranthus duckei*. It is distinct from the latter primarily by the larger fruit. Moreover, it is reported to be a tall tree (without more precise indication of size), whereas reports for *Tetrameranthus duckei* indicate a medium-sized tree to about 12 m tall so far. Nevertheless one might wonder if *Tetrameranthus macrocarpus* is merely an extreme form of *Tetrameranthus duckei*, but more extensive collecting in the western Amazon region is needed before this question could be answered.

### Tetrameranthus pachycarpus

Westra

http://species-id.net/wiki/Tetrameranthus_pachycarpus

Fig. 1E, F Map 1

Tetrameranthus pachycarpus Proceedings of the Koninklijke Nederlandse Akademie van Wetenschappen, ser. C. 88: 477, plate 2, fig.1, plates 12 & 13. 1985.

#### Type.

*Klug 1216* (holotype NY; isotypes F, U), Peru, Loreto: Mishuyacu, near Iquitos, 100 m, April 1930.

#### Description.

*Tree*, 4–26 m tall, young twigs and petioles densely to rather densely covered with brown, stellate hairs >0.5 mm long, becoming glabrous. *Leaves*: petioles 20–30 mm long, 2–4 mm diam., slightly thickened toward the base, lamina elliptic, narrowly elliptic or narrowly obovate, 17–22 by 5–10 cm (index 2.1–3.4), coriaceous, brown in sicco, glabrous except for primary vein above, rather densely to sparsely covered with stellate hairs on large veins and otherwise sparsely covered with stellate hairs to glabrous below, base acute to attenuate, apex obtuse, acute, or acuminate (acumen 1–5 (rarely more) mm long), primary vein impressed to almost flat above, secondary veins 10–15 on either side of primary vein, not loop-forming, or less often loop-forming, shortest distance between loops and margin 2–3 mm, tertiary veins flat and inconspicuous above, percurrent to more or less reticulate. *Inflorescences* 1-flowered; peduncles 4–5 mm long, 1–2 mm diam., fruiting peduncles to c. 5 mm diam., bracts 2(–more?), narrowly triangular to linear-triangular, 3–5 mm long, outer side densely covered with stellate hairs, falling at or after flowering, pedicels 10–15 mm long, 1.5–2 mm diam., fruiting pedicels to c. 25 mm long, 3–4 mm diam., peduncles and pedicels densely to rather densely covered with stellate hairs, becoming glabrous; flowers yellow in vivo; sepals broadly elliptic, 3–4 mm long, connate at the very base, outer side densely covered with stellate hairs; outer petals narrowly elliptic to oblong, to c. 35 by 16 mm, outer side densely to rather densely covered with stellate hairs, the inner base glabrous, to c. 3 mm long, inner petals narrowly elliptic to oblong, somewhat smaller than outer petals and with slightly larger glabrous inner base; stamens c. 2 mm long, connective shield more or less conical and curved toward the center. *Monocarps* 1–3, yellow in vivo, brown in sicco, ellipsoid, 2-seeded forms to c. 70 by 40 mm, without or with inconspicuous, oblique constriction, apex rounded. *Seeds* 1–2 per monocarp, 30–40 by 20–28 mm.

#### Distribution.

Peru (Loreto), fairly common in the region around Iquitos, not known elsewhere so far.

#### Habitat and ecology.

In forest on white sand. At elevations of 100–200 m. Flowering and fruiting: probably throughout most of the year.

#### Additional specimens examined.

**Peru.** Loreto: Mishana, 100–140 m, Ayala 1564 (AMAZ), Díaz S. et al. 404 (MO), Foster 4271 (F, MO, U), Gentry et al. 39313 (U), Vásquez et al. 5285 (MO, U), Vásquez & Jaramillo 9651 (MO, U); Puerto Almendras, 100–120 m, Díaz & Arévalo 81 (MO, U), Grández & Jaramillo 4983 (MO, U), Vásquez & Jaramillo 4619 (MO, U), 11031 (MO, U), Vásquez et al. 8067 (MO, U); Carretera de Peña Negra, at 2 km from Quista Cocha, 180 m, Rimachi Y. 4537 (MO, US), 7735 (MO, NA); Ninarumi (“Nina Rumy”), 123 m, Ruiz M. 808 (MO); Prov. Maynas, Allpahuayo, Vásquez et al. 17984 (MO).

### Tetrameranthus umbellatus

Westra

http://species-id.net/wiki/Tetrameranthus_umbellatus

Figs 1A, B, G 2F, G Map 2

Tetrameranthus umbellatus Proceedings of the Koninklijke Nederlandse Akademie van Wetenschappen, ser. C. 88: 479, plates 1, 14, 15. 1985.

#### Type.

*Tunquí 62* (holotype U; isotype MO), Peru, Amazonas: Río Santiago, Huambisa, other side of La Poza 1 km, 180 m, 14 November 1979.

#### Description.

*Tree*, 8–25 m tall, 15–25 cm diam., young twigs and petioles densely to rather densely covered with stellate hairs <0.2 mm long, becoming glabrous. *Leaves*: petioles 5–25 mm long, 1.5–3 mm diam., lamina narrowly obovate or narrowly elliptic-obovate, 15–30 by 4–9 cm (index 2.4–4.7), chartaceous, greenish brown to brown above, pale greenish brown to brown below in sicco, sparsely covered with stellate hairs to glabrous above, rather densely to sparsely covered with stellate hairs on primary vein and sparsely covered to glabrous otherwise below, base acute to attenuate, decurrent along the petiole, apex acute to (abruptly) acuminate (acumen 5–10 mm long), primary vein flat to slightly impressed above, secondary veins 13–20 on either side of primary vein, slightly impressed to raised above, loop-forming, shortest distance between loops and margin 0.5–2 mm, tertiary veins flat to raised above, percurrent to more or less reticulate. *Inflorescences* up to 5-flowered, umbel-like; peduncles 5–25 mm long, c. 1.5 mm diam., densely to rather densely covered with stellate hairs, becoming glabrous in age, fruiting peduncles to c. 3 mm diam., bracts 4, oblong, 4–5 mm long, outer side densely covered with stellate hairs, falling before flowering, pedicels 25–70 mm long, ≥1 mm diam., densely to rather densely covered with stellate hairs, becoming glabrous in age, fruiting pedicels to c. 3 mm diam. *Flowers* green, maturing yellow in vivo; sepals broadly triangular-ovate to ovate, connate just at the base, to c. 4 by 3–5 mm, outer side densely covered with stellate hairs; outer petals ovate-elliptic, 16–20 by 9–12 mm, densely to rather densely covered with stellate hairs, the inner base with small glabrous area, inner petals elliptic, 13–17 by 6–7 mm, indument as in outer petals, the glabrous inner base slightly larger; stamens ≤1 mm long, connective shield cushion-shaped, flat. *Monocarps* 2–7, orange or red in vivo, brownish black in sicco, ellipsoid to oblongoid, 2-seeded forms to c. 45 by 20 mm and with oblique constriction, 1-seeded forms smaller and without constriction, mostly rounded at the apex. *Seeds* 1–2 per monocarp, to c. 25 by 18 mm.

#### Distribution.

Amazonian Peru (Amazonas, Huánuco, Loreto) and Brazil (Amazonas, Pará).

#### Habitat and ecology.

In non-inundated (terra firme) forest or rarely periodically inundated forest. At elevations of 100–600 m. Flowering and fruiting: probably throughout the year.

#### Additional specimens examined.

**Brazil.** Amazonas: Mun. Jutaí, Cid et al. 7269 (K, NY, US); km 1225 of Cuiabá-Santarém Highway (BR 163), Prance et al. 25602 (NY, U). **Peru.** Amazonas: Río Santiago, 2 km below Caterpiza, trail to Mitayar, E side of Quebrada Caterpiza, Huashikat 613 (MO, U); Quebrada Sasa, Monte Numi, 600 m, Kayap 2015 (MO, U). Huánuco: Distr. Llullapichis, Prov. Puerto Inca, Dantas, 280 m, Kröll Saldaña 712 (U); S of Pucallpa, next to the junction of the Río Pachitea and Río Llullapichis, 260 m, Morawetz & Wallnöfer 14-81085 (U); Prov. Pachitea, region of Pucallpa, Sira Mts., 26 km S of Puerto Inca, next to the junction of the Río Pachitea and Río Llullapichis, field station “Panguana”, 260 m, Morawetz & Wallnöfer 18-14188 (U, WU). Loreto: Prov. Maynas, Paucarillo Reserve, Río Amazonas, 110 m, Choo 347 (MO); Prov. Maynas, Distr. Las Amazonas, Explornapo Camp, near Sucusari, 100–140 m, Pipoly et al. 14565 (MO, U); Prov. Maynas, Llachapa, Río Napo, 130 m, Vásquez & Jaramillo 3732 (MO, U).

#### Vernacular names.

Peru: Washi yais (*Tunquí 62*), Wáshi yéis (*Huashikat 613*), Yaú (*Kayap 2015*).

## Supplementary Material

XML Treatment for Tetrameranthus

XML Treatment for Tetrameranthus duckei

XML Treatment for Tetrameranthus globuliferus

XML Treatment for Tetrameranthus guianensis

XML Treatment for Tetrameranthus laomae

XML Treatment for Tetrameranthus macrocarpus

XML Treatment for Tetrameranthus pachycarpus

XML Treatment for Tetrameranthus umbellatus
